# Grape Pomace Extract-Loaded Liposomes Enriched Cream Formulations for Skincare

**DOI:** 10.3390/antiox15040421

**Published:** 2026-03-27

**Authors:** Cristiana Radulescu, Radu Lucian Olteanu, Ramona-Daniela Pavaloiu, Fawzia Sha’at, Gabriela Stanciu, Mihaela Nechifor (Tudorache)

**Affiliations:** 1 Faculty of Sciences and Arts, Valahia University of Targoviste, 13 Sinaia Alley, 130004 Targoviste, Romania; cristiana.radulescu@valahia.ro; 2 Doctoral School Chemical Engineering and Biotechnology, National University of Science and Technology Politehnica of Bucharest, 313 Splaiul Independenței, 060042 Bucharest, Romania; tudorache.mihaela-db@ansvsa.ro; 3 Academy of Romanian Scientists, 3 Ilfov, 050044 Bucharest, Romania; 4 Synthesis of Bioactive Substances and Pharmaceutical Technologies Department, National Institute for Chemical-Pharmaceutical Research and Development—ICCF, 112 Vitan Avenue, 3rd District, 031299 Bucharest, Romania; fawzya.shaat@gmail.com; 5 Department of Chemistry and Chemical Engineering, Faculty of Applied Sciences and Engineering, Ovidius University of Constanta, 900527 Constanta, Romania; gstanciu@univ-ovidius.ro

**Keywords:** grape pomace extract, liposome, cream formulations, antioxidant activity, polyphenol release

## Abstract

This study aims to develop and characterize novel dermatocosmetic formulations designed to hydrate the skin, improve its appearance, reduce wrinkles, and provide antioxidant, anti-ageing, antimicrobial, and anti-inflammatory benefits, along with potential protection against UVA and UVB radiation. The formulations contain the following ingredients: xanthan gum (0.5%), *Calendula officinalis* oil (5%), *Argania spinosa* oil (5%), *Helianthus annuus* oil (5%), liposomes containing a hydroalcoholic extract of pomace from local red or white grapes (2%), an olive oil-based emulsifier (6%), vitamin E (0.5%), cetearyl alcohol (3%), propylene glycol (8%), and purified water (up to 100%). The natural ingredients used in these formulations, i.e., the red or white grape pomace extract from the aforementioned Romanian varieties, the oils of *Calendula officinalis*, *Argania spinosa*, and *Helianthus annuus*, xanthan gum, and the olive oil-based emulsifier (Olliva), promote the concept of ‘green cosmetics’. The use of liposomes to deliver bioactive substances from hydroalcoholic extracts allows the gradual release of active ingredients into the skin. An alternative for incorporating grape pomace extract into a cream-type matrix involves the use of liposomes. Liposomes loaded with red or white grape pomace extract were prepared using the thin-film hydration technique, followed by ultrasonication and extrusion. The obtained formulations were characterized using bio-physico-chemical analysis procedures in terms of consistency, colour, homogeneity, aroma, pH, stretch, texture, stability, and antioxidant activity/free radical scavenging capacity, as well as in vitro polyphenol release behaviour. These newly developed dermatocosmetic formulations were the subject of a patent application in Romania.

## 1. Introduction

The transformation of winemaking by-products into natural therapeutic agents is a crucial aspect of sustainable development and eco-friendly waste management. Recent advances have broadened the use of extracts from grape pomace, especially in the cosmetics and food industries, thanks to the highly valued bioactive compounds present in these solid residues, even after the production of final products [1,2].

Promoting the new concept of ‘green cosmetics’ means emphasizing cosmetic products that are made with natural, ethical, and sustainable ingredients that respect the health of consumers and the environment. Rich in polyphenols, flavonoids, antioxidants, and fibres, grape pomace can be transformed into natural active ingredients, such as extracts, for cosmetic products using environmentally friendly ‘green extraction methods’ [3]. These extracts can be incorporated into creams, lotions, or serums and have antioxidant, anti-ageing, protective, and moisturizing effects [3,4,5].

Grape pomace, a by-product of grape pressing in the food industry, is a valuable source of polyphenols, dietary fibre, natural sugars, vitamins, and seed oils [6,7]. Research has highlighted its potential for multiple applications in various sectors, including the food industry (e.g., functional products and nutritional supplements), cosmetics (e.g., antioxidant extracts and regenerating oils), energy (e.g., bioethanol and biogas), and agriculture (e.g., compost and cultivation substrates) [8,9,10,11,12]. Grape pomace from red and white *Vitis vinifera* L. varieties brings an important amount of natural antioxidants due to its high phenolic compound content. Approximately 70% of these compounds are found in the solid residue [12], which consists of two fractions: seedless grape pomace (residual pulp, skin, and bunches) and seeds. Both fractions are rich in bioactive compounds, including phenolic compounds. Grape pomace generally contains skin, pulp, seeds, and residual bunches, representing around 25% of the total weight of grapes used in the winemaking process [10]. Grape skin, pulp, and seeds are known to contain significant amounts of polyphenols in both fresh and extracted forms, primarily flavonoids (such as anthocyanins, flavonols, flavan-3-ols, flavones, and chalcones) and non-flavonoids (such as phenolic acids, stilbenes, tannins, coumarins, and neolignans) [13]. Red grape skin extracts contain much higher amounts of the antioxidant compounds resveratrol, quercetin, and rutin than extracts obtained from other parts of the grape. Red grape pomace has also been shown to have appreciable antioxidant capacity. Beres et al. (2017) highlighted the potential to recover phenols and antioxidant fibres from the skin and seed oil of the pomace [10]. The most abundant phenolic compounds in grape pomace are anthocyanins, which are concentrated in the skin, and flavonols, which are especially present in the seeds (56–65% of the total) [14].

These phenolic compounds are valuable secondary metabolites with antioxidant properties, and the total content of these compounds in grape pomace extracts is well correlated with their antioxidant activity [15]. Grape pomace extracts are currently used in pharmaceutical and cosmetic products in liquid, concentrated, or powdered form. Due to their antioxidant capacity, these extracts have also been used to prevent lipid oxidation in fish-based products and exhibit antimicrobial activity. Also, the antimicrobial effect of grape seed products is generally attributed to various phenolic compounds. Garcia-Lomillo et al. revealed that phenolic acids play a more significant role than flavonoids, with gallic acid, *p*-hydroxybenzoic acid, and vanillic acid being the most prevalent [14].

Polyphenols are ubiquitous in the plant kingdom, and dozens of epidemiological studies have established a clear link between their presence and reduced oxidative stress, inflammation, and ageing. This suggests that they may play an important role in supporting solar photoprotection in the epidermis. In recent years, cosmetic research has focused on natural plant-derived bioactive compounds as alternatives to synthetic ones [16]. Polyphenols are one such group of compounds that have been studied in depth in recent decades. Understanding the biological and bioactive role of polyphenols as antioxidants and anti-inflammatory agents in pharmaceutical applications and dermatological therapy is important, as is highlighting their main mechanisms of action and structure–activity relationship [16,17,18].

To increase stability and prevent the compounds from oxidizing and degrading during storage, they can be encapsulated in liposomes [19]. Encapsulation in liposomes improves the stability of plant extracts against environmental factors due to the high concentration of polyphenols and the controlled oxygen levels [20].

Several studies [21,22] emphasize their unique capacity to encapsulate both hydrophilic and lipophilic compounds simultaneously, which is crucial for the controlled and targeted delivery of active ingredients, including vitamins, peptides, coenzyme Q10, hyaluronic acid, and antioxidants. These properties are particularly valuable in dermatocosmetics, where an ingredient’s success depends on its ability to cross the skin barrier. Studies on the interaction of liposomes with the skin [23] demonstrate that these lipid vesicles can fuse with the corneocyte layers, facilitating the penetration of active substances into the epidermis and dermis. The size of liposomal particles also directly influences cutaneous diffusion efficiency [24]: particles below 200 nm are associated with increased permeability.

Liposomes are beneficial in dermatocosmetics for stabilizing light- and oxidation-sensitive molecules preventing degradation, and gradually releasing active ingredients to reduce irritation and improve tolerability [25,26]. Squizzato et al. revealed that the phospholipids used in their composition are compatible with the skin and actively contribute to restoring the lipid barrier, thereby improving skin hydration and elasticity [27]. Safety assessments show minimal risk at cosmetic concentrations [28], while Shaat et al. [29] highlight liposomes as advanced ‘vectorisation’ tools, enabling more efficient, faster-acting dermatocosmetic products. Optimizing phospholipid properties enhances ingredient penetration, stability, and release. From a scientific standpoint, liposomes are an efficient and safe technology for delivering active ingredients, enhancing compound stability, increasing skin permeability, and optimizing ingredient release to improve skin barrier function.

In developing the dermocosmetic field and promoting the concept of ‘green cosmetics’, a key goal is to create products with complex efficacy based on mechanisms that regulate skin tissue function. Dermocosmetics are designed to combine esthetic benefits with functional effects on skin health. Unlike traditional cosmetics, which primarily cleanse, scent, or beautify, dermocosmetics contain bioactive compounds (e.g., antioxidants, vitamins, hyaluronic acid, etc.) that interact with cellular and molecular pathways in the skin to improve hydration, barrier function, or signs of ageing [30,31,32]. Dermocosmetics are legally classified as cosmetics under European Union law, specifically Regulation (EC) No 1223/2009 [33], provided they do not claim to treat, prevent, or cure disease; any therapeutic claim would reclassify the product as a medicinal product under Directive 2001/83/EC [34]. Dermocosmetics thus occupy a unique niche at the interface of dermatology and cosmetology, relying on scientifically validated active ingredients to enhance skin function while remaining within the legal framework for cosmetic products. In this context, grape-derived polyphenols can be incorporated into dermocosmetic formulations, leverageing their antioxidant and anti-aging properties within the regulatory framework for cosmetic products.

One of the goals of this research is to propose a feasible solution using active compounds from various plant species, including grape-based extracts or products derived from their processing, to further utilize their biological properties. A second objective is to obtain a liposome-based cream for dermatocosmetic use. This cream should be produced with natural, sustainable ingredients to promote the concept of ‘green cosmetics’. It should hydrate the skin and improve its appearance, prevent the formation of wrinkles, and have anti-ageing, antimicrobial, anti-inflammatory, and protective effects against UVA and UVB radiation. Liposomal encapsulation protects polyphenolic compounds from environmental degradation by shielding them from oxidation and light exposure. Furthermore, liposomes can enhance skin penetration and ensure the sustained release of active ingredients, ultimately improving the formulation’s biological efficacy.

## 2. Materials and Methods

### 2.1. Materials and Reagents

The reagents and natural materials used in this research were of high purity. They included xanthan gum, propylene glycol, vitamin E, phosphatidylcholine, and phosphate-buffered saline (PBS) buffer solution (0.1 M, pH 7.4) (all from Sigma-Aldrich, Merck Group, Darmstadt, Germany). *Calendula officinalis* (marigold) and *Argania spinosa* oils, as well as Olliva emulsifier, were purchased from Mayam Elemental SRL in Romania. *Helianthus annuus* (sunflower) oil (Pure Body Naturals, Cincinnati, OH, USA), cetearyl alcohol, and ethanol (BLD Pharmatech, Shanghai, China) were also used. Furthermore, 2,2-diphenyl-1-picrylhydrazyl (Tokyo Chemical Industry (TCI), Tokyo, Japan) was used to investigate antioxidant activity. The following materials were used to obtain enriched cream formulations with grape pomace extract-loaded liposomes: xanthan gum (0.5%), propylene glycol (8%), *Calendula officinalis* oil (5%), *Argania spinosa* oil (5%), *Helianthus annuus* oil (5%), an olive oil-based emulsifier (6%), liposomes containing a hydroalcoholic extract of grape pomace (autochtonous varieties: red, e.g., Fetească Neagră and Negru de Drăgășani; and white, e.g., Fetească Albă and Tamâioasă Românească) (2%), vitamin E (0.5%), cetearyl alcohol (3%), and ultrapurified water (up to 100%).

### 2.2. Obtaining the Hydroalcoholic Grape Pomace Extracts by Ultrasonication

The hydroalcoholic extract of pomace is obtained from red grapes (Fetească Neagră, code FN, and Negru de Drăgășani, code ND) and white grapes (Fetească Albă, code FA, and Tămâioasă Romanească, code TR) according to the procedure described by Ivanova et al. (2011) [35], and adapted by Radulescu et al. (2024) [12], in previous research. Briefly, the working parameters used to obtain the extracts are an extraction ratio of dry plant material: solvent (g:cm^3^) = 1:5; solvent for extraction: 70% ethanol. The obtaining of hydroalcoholic extracts by ultrasonication using VWR^®^ Ultrasonic Cleaner USC 200 TH system (VWR International LLC, Radnor, PA, USA), followed the next steps: (*i*) 1 g of lyophilised plant material is weighed directly into 15 mL bottles with a cap; (*ii*) 5 mL of solvent is added; (*iii*) extraction is carried out by ultrasonication for 20 min (initial temperature 29 °C and final temperature 39 °C, frecvency 50 Hz) with intermittent agitation (vortex) every 5 min; (*iv*) the supernatant is separated by centrifugation at room temperature (10 min/6000 rpm); (*v*) the supernatant is collected; (*vi*) 5 mL of solvent is added to the precipitate in the bottle and extraction is repeated by ultrasonication followed by separation by centrifugation; (*vii*) the supernatants obtained in steps (iv) and (vi) are reunited; (*viii*) the extracts are stored at 4 °C for subsequent analysis. The final extract obtained was characterized using biophysicochemical analysis procedures reported by Radulescu et al. in previous studies [12,20,36].

### 2.3. Preparation of Liposomes Containing White and Red Grape Pomace Extracts

Liposomes loaded with red and white grape pomace extracts (coded L@ND, L@FA, L@FN, and L@TR) were prepared using the thin-film hydration method, followed by sonication and extrusion. The lipid phase was prepared by dissolving 100 mg of phosphatidylcholine in 10 mL of ethanol and then adding 0.75 mL of the extract. The mixture is then left to stand at 25 °C overnight to allow the phospholipids to swell. The solvent is then removed using a Laboranta 4000 rotary evaporator (Heidolph Instruments GmbH & Co. KG, Kelheim, Germany) under reduced pressure at 37 °C for two hours until a thin lipid film forms. This film is then hydrated with distilled water at 37 °C, and the resulting dispersion is stored at 25 °C for two hours to stabilize the liposomes. To reduce the size of the vesicles, the suspension is sonicated for 20 min at 50% amplitude in a Sonorex-Digital-10P ultrasonic bath (Bandelin-Electronic, Berlin, Germany), one minute on, one minute off, followed by sequential extrusion through polycarbonate membranes with pore sizes of 0.4 µm and 0.2 µm five times each. The liposomes are then separated from the unencapsulated extract by centrifugation at 10,000 rpm and 5 °C for 20 min. The pellet containing the loaded liposomes is redispersed in distilled water. Samples were prepared in triplicate and stored at 4 °C until further analysis.

#### Characterization of Liposomal Formulations

Liposomal formulations (i.e., L@ND, L@FA, L@FN, and L@TR) are characterized based on encapsulation efficiency (EE), defined as the ratio of polyphenols encapsulated in the liposomes to the total polyphenol content of the initial extract.

The total amount of polyphenols entrapped in liposomes was assessed using the Folin–Ciocalteu method as described in several papers [19,28,37]. To determine the EE, the liposomes (1 mL) were mixed with 0.5 mL of 0.5% Triton X-100 and vortexed to break down the lipid membranes. The suspension was diluted ten times with methanol and then filtered. In short, 1 mL of liposomes containing extract was mixed with 10 mL of water and 1 mL of Folin–Ciocalteu reagent (10× diluted). The mixture was then made up to 25 mL with a 5% (wt) aqueous sodium carbonate solution. After 30 min, the absorbance of the solution was measured at 760 nm using a UV/VIS spectrophotometer (Helios λ, ThermoFisher Scientific, Waltham, MA, USA). The total polyphenol content was calculated from a calibration curve of gallic acid (0.01–0.1 mg/mL concentration range; y = 0.0132x + 0.027, R^2^ = 0.9968). The results were expressed as milligrams of gallic acid equivalents per gram of dry extract (mg GAE/g). Empty liposomes were used as a control. For the pomance extract-loaded lipid vesicles, particle size and polydispersity index (PDI) were evaluated. Particle size and PDI were determined by dynamic light scattering (DLS) using a particle analyzer. To minimize multiple-scattering effects, the liposomal dispersions were diluted with distilled water at a 1:10 ratio prior to analysis. All measurements were conducted at room temperature.

### 2.4. Formulation of Dermatocosmetic Formulations Enriched with Liposome Oil/Water (O/W)

Creams enriched with liposomes, coded Cream-L@ND, Cream-L@FA, Cream-L@FN, Cream-L@TR, containing red and white grape pomace extracts, were prepared using the composition in Table 1. The oily phase components (*Calendula officinalis* oil, *Argania spinosa* oil, *Helianthus annuus* oil, vitamin E, cetearyl alcohol, and Olliva emulsifier) are mixed and heated in a water bath at 70 °C for 15 min. The xanthan gum is dispersed in water and left to swell completely for 30 min. Propylene glycol is then added. The components of the aqueous phase were mixed and heated for 15 min at 70 °C to form the aqueous phase. After heating, the oil phase is gradually added to the aqueous phase under continuous mixing until a uniform, smooth paste is obtained. Liposomes with extracts are then added once the mixture has cooled to 40 °C. Also, we prepared a base cream with no liposomes or white grape pomace extracts (coded Cream-Base).

The creams were packaged in amber glass containers and stored in a refrigerator at 4–8 °C until further use. The cream’s potential protective properties against UVA and UVB radiation, as well as its antimicrobial and anti-inflammatory properties, are derived from its natural ingredients, including marigold, argan, and sunflower oils. Marigold oil in particular has a soothing and healing effect. The cosmetic formulations were characterized using biophysical and chemical analyses to assess consistency, colour, homogeneity, flavour, pH, spreadability, texture, stability, and antioxidant activity/free-radical scavenging capacity.

#### 2.4.1. Visual Appearance

A visual examination was performed to check for consistency, colour, homogeneity, and flavour. A small amount of each formulation, i.e., grape pomace extract-loaded liposomes (L@FN, L@ND, L@FA, L@TR) enriched cream (Cream-L@ND, Cream-L@FA, Cream-L@FN, Cream-L@TR), was placed on a glass slide and examined using a magnifying glass with 4.5× magnification. This initial inspection ensured the uniformity and stability of the formulations and allowed for the identification of any visible irregularities in texture or Colour that might indicate instability.

#### 2.4.2. pH Analysis

The pH of the formulations (Cream-L@ND, Cream-L@FA, Cream-L@FN, Cream-L@TR) was measured with a digital pH meter (Mettler Toledo, Columbus, OH, USA) after the samples had been completely homogenized. Measurements were taken after allowing the reading to stabilize for 30 s. Three separate readings were recorded for each sample at different points. If the variation between values exceeded ±0.2 pH units, the process was repeated to confirm accuracy. This analysis is crucial for confirming the formulation’s skin compatibility, as pH plays a key role in maintaining skin barrier function.

#### 2.4.3. Spreadability Evaluation

The spreadability test was performed using the modified Ojeda-Arbussa method. 1 g of each sample was set among two plates (for 1 min each). A series of weights applied at one-minute intervals was used to generate cumulative loads of 175 g, 225 g, 275 g, 325 g, 625 g, and 875 g. The weights ranged from 50 g to 750 g. The tensile diameter was measured after each load; the spreading area (*Si*) was calculated using Equation (1).

(1) $$S_{i}=d_{i}^{2}\left(\frac{\pi }{4}\right)$$

where *S_i_*—spreading surface area (mm^2^) for the applied load *i* (g); *d_i_*—mean diameter (mm) of the sample under load.

#### 2.4.4. Texture Profile Analysis

The cream texture profile analysis (TPA) was performed using a TX-700 texture analyser (Lamy Rheology, Champagne-au-Mont-d’Or, France), equipped with a 10 kg force sensor. A two-step compression test was performed in terms of mimicking product application. The test was used to determine: (i) firmness, defined as the maximum force required for initial deformation; (ii) cohesion, which is the energy ratio between successive compressions and is a measure of the structural integrity of the gel; (iii) elasticity, defined as the ability of the cream to regain its shape after deformation. A 30 g sample of grape pomace extract-loaded liposomes enriched cream (Cream-L@ND, Cream-L@FA, Cream-L@FN, Cream-L@TR) was placed in a cylindrical container with levelled surfaces, and the test used a hemispherical probe. The compression rate was set at 0.8 mm/s, the deformation depth to 10 mm, the trigger force to 5 g (0.05 N), and the interval between compressions to 5 s. All tests were carried out at room temperature to replicate the application to the skin. To ensure reproducibility, triplicate analyses were performed.

#### 2.4.5. Stability Monitoring

Over 60 days, the stability of the formulations was monitored. The samples were stored in brown-walled glass containers at 25 °C. The visual appearance, pH, and flavour were re-evaluated after this time to detect potential signs of degradation or instability, such as phase separation, colour changes, or odour alterations.

#### 2.4.6. DPPH Free Radical Scavenging Method

The antioxidant activity of the liposome-based creams (i.e., Cream-L@ND, Cream-L@FA, Cream-L@FN, and Cream-L@TR) was evaluated using the DPPH free radical scavenging method. This method is used to assess the preservation of bioactive compounds after formulation. Extraction was performed in ethanol (i.e., 1 g:10 mL solvent), then the obtained mixture was stirred for five minutes and filtered twice. Next, 0.6 mL of this solution was mixed with 2.4 mL of a DPPH solution in ethanol (0.025 g/L). The samples were then incubated in the dark for 30 min, at 25 °C. The DPPH radical’s ability to absorb light at 517 nm was measured using a Jasco V-630 UV-Vis spectrophotometer (Portland, OR, USA).

The antioxidant activity was calculated using Equation (2) and a calibration curve with Trolox as the standard:

(2) AA (%) = [(A_DPPH_ − (A_Sample_ − A_blank_))/A_DPPH_] × 100

where A_DPPH_ represents the absorbance of the control solution. A_Sample_ is the absorbance of the reaction mixture containing the DPPH solution. A_blank_ is the absorbance of the cream mixed with the solvent (without DPPH).

The Trolox calibration curve was prepared by measuring the DPPH radical-scavenging activity of Trolox solutions at concentrations ranging from 5 to 50 μM under the same experimental conditions. The calibration curve for Trolox, used to quantify antioxidant activity via the DPPH assay, was given by the equation: y = 51.703x + 1.691, with a coefficient of determination (R^2^) of 0.9964, demonstrating excellent linearity within the tested concentration range. Results were expressed as both percentage inhibition and Trolox equivalent antioxidant capacity (mM Trolox/g cream).

### 2.5. In Vitro Polyphenols Release Behaviour

The release behaviour of polyphenols from the liposome-based dermatocosmetic creams (i.e., Cream-L@ND, Cream-L@FA, Cream-L@FN, Cream-L@TR) was assessed using a Franz diffusion cell system (Orhid Scientific and Innovative Pvt Ltd., Nashik, India) designed to measure in vitro drug release from creams. The donor compartment was filled with a 0.5 g sample, and the receptor compartment was filled with 100 mL of PBS to simulate physiological conditions. The diffusion cells were maintained at 32 °C (the skin temperature) and continuously stirred at 100 rpm to ensure the receptor phase was fully homogeneous. Samples were extracted from the receptor medium at the following intervals: 15, 30, and 45 min; 1, 2, 3, 4, and 5 h; and 1, 2, 3, 4, and 5 days. A small amount of the liquid was taken from the container, and an equal amount of fresh PBS was added to keep the same conditions for absorption. The amount of polyphenols in each sample was measured using UV-Vis spectrophotometry (Jasco V-630, Portland, OR, USA).

## 3. Results and Discussion

The liposome-based dermatocosmetic formulations have the compositions presented in Table 1, briefly: xanthan gum 0.5%, propylene glycol 8%, *Calendula officinalis* oil 5.0%, *Argania spinosa* oil 5%, *Helianthus annuus* oil 5%, liposomes with hydroalcoholic extract of red/white grape pomace 2%, vitamin E 0.5%, cetearyl alcohol 3%, olive oil-based emulsifier 6%, and purified water up to 100%. The natural ingredients, namely red/white grape pomace extracts, *Calendula officinalis*, *Argania spinosa*, and *Helianthus annuus* oils, xanthan gum, and olive oil-based vegetable emulsifier, promote the current concept of “green cosmetics”. Using liposomes to deliver bioactive substances from hydroalcoholic extracts results in the active principles being progressively released into the skin. In this regard, this research aimed to develop liposomal formulations that would hydrate the skin, improve its appearance, reduce wrinkles, and provide anti-ageing, antimicrobial, anti-inflammatory, and potentially protective effects against UVA and UVB radiation.

### 3.1. Characterization of Liposomes Encapsulated Red and White Grape Pomace Extracts

Liposomal encapsulation protects polyphenolic compounds from environmental degradation by shielding them from oxidation and exposure to light. Additionally, liposomes can enhance skin penetration and deliver active ingredients over an extended period, ultimately improving the formulation’s biological efficacy. The thin-film hydration technique, followed by sonication and extrusion, is an easy, consistent, and widely applicable method for preparing liposomes, offering good control over size, versatility in encapsulation, and compatibility with a wide range of bioactive extracts.

The entrapment efficiency of liposomes loaded with local red and white grape pomace extracts (coded L@ND, L@FA, L@FN, and L@TR) ranged from 65.62 ± 0.06% to 60.12 ± 0.05% (Table 2). The variation in EE could be linked to the physicochemical properties of the extracts (e.g., polarity, molecular weight), which influence interaction with the lipid bilayer during hydration. The mean particle sizes of the liposomal formulations ranged from 166.3 nm to 184.2 nm, indicating the formation of nanometric vesicles suitable for dermatocosmetic applications.

The polydispersity index (PDI) values ranged from 0.301 to 0.350, indicating a moderately narrow size distribution and good overall homogeneity among the liposome populations (Table 2). All samples presented PDI values below 0.4, which is typically acceptable for stable colloidal systems intended for topical use. PDI is used as an indicator of the extent of the molecular weight distribution. Similar results were previously reported. For example, Montagner et al. (2022) [38] reported grape seed extract liposomes via the reverse-phase evaporation method with a 239 nm and PDI of 0.252, and Rached et al. (2025) [39] prepared liposomes containing a Lebanese grape variety via hydration combined with sonication with a size of 148 nm and a PDI value below 0.3.

Therefore, liposomes exhibited appropriate nanoscale dimensions, good homogeneity, and satisfactory encapsulation efficiencies, indicating that all formulations are well-suited for incorporation into dermatocosmetic creams, ensuring effective delivery and stability of the pomace-derived actives.

### 3.2. Characteristics of Dermatocosmetic Formulation

Red and white grape pomace extracts from the local Fetească Neagră and Negru de Drăgășani varieties, and Fetească Albă and Tămâioasă Românească varieties, respectively, are embedded in liposomes and harmonized with a series of natural essential oils, such as *Calendula officinalis*, *Argania spinosa*, and *Helianthus annuus*, xanthan gum, Olliva emulsifier, and vitamin E. These ingredients form a creamy, odourless, pearly white-yellow dermatocosmetic formulation (Table 3). Additionally, a Cream-base was prepared as a reference formulation. Cream-base consists of the same excipients (xanthan gum, Olliva emulsifier, and vitamin E) but lacks liposomes or white grape pomace extracts liposome, serving to highlight the impact of the extracts on the cream’s physical, sensory, and functional properties.

Thus, the dermatocosmetic creams enriched with liposomes (i.e., Cream-L@ND, Cream-L@FA, Cream-L@FN, Cream-L@TR) demonstrated consistent physical and sensory characteristics. Organoleptic evaluations after 60 days at 4–8 °C of storage confirmed the stability of all formulations, with each cream maintaining a homogeneous, pearl-yellowish-white appearance, odourless profile, and no signs of phase separation, sedimentation, or texture degradation. The pH values remained within a physiologically acceptable dermal range (5.01–5.37), showing only minimal fluctuations over time, indicating the formulations’ chemical stability (Table 3). Cream-base exhibited similar organoleptic stability, maintaining homogeneity and odourless appearance after 24 h, with pH 5.25 ± 0.01, slightly higher than the liposome-enriched creams but still within the acceptable dermal range (Table 4). The slightly higher pH may be attributed to the absence of acidic phenolic compounds from grape pomace extracts, which can lower the pH of enriched formulations, as previously reported in cosmetic creams enriched with grape stem extracts [9]. This confirms that the base formulation itself is stable and compatible with skin pH.

The texture analysis profile (TPA) of dermatocosmetic creams is a critical parameter that reflects the mechanical and sensory properties of a formulation, directly influencing its consumer acceptance, stability, and performance on the skin. Parameters such as firmness, cohesiveness, and springiness provide quantitative insights into the product’s structure, spreadability, and resilience during application. At 24 h, Cream-L@FA exhibited the highest firmness (0.500 N), followed closely by Cream-L@TR and Cream-L@ND, whereas Cream-L@FN showed the lowest value (0.394 N). The Cream-base exhibited lower firmness (0.378 N) than the liposome-enriched formulations, highlighting the reinforcing impact of both liposome incorporation [40] and bioactive grape extracts on the structural integrity of the creams, as previously observed in grape-extract-enriched cosmetic formulations [9]. Cohesiveness and springiness displayed similar patterns, with Cream-L@FN presenting the greatest cohesiveness (0.598) and Cream-L@FA the highest springiness (0.997), indicating enhanced structural recovery and elasticity in these formulations (Table 4 and Table 5). By comparison, Cream-base cohesiveness (0.572) and springiness (0.820) were slightly lower, suggesting that the presence of liposome-loaded pomace extracts enhances the overall structural strength and elasticity of the matrix, in agreement with previous reports on nanomaterial- and grape-extract-enriched cosmetic formulations [9,40]. After 30 days, all samples demonstrated an overall increase in TPA parameters, particularly firmness, with Cream-L@FA and Cream-L@ND reaching 0.801 N and 0.753 N, respectively (Table 4 and Table 5). Although Cream-base data after 30 days were not measured, its lower initial firmness suggests that matrix strengthening would be less pronounced without active extracts.

This behaviour suggests a progressive strengthening of the formulation matrix, likely resulting from polymeric interactions (e.g., xanthan gum cross-linking) or internal structural rearrangements during storage. Cohesiveness values became more uniform among formulations at this stage, stabilizing between 0.46 and 0.50, while springiness exhibited a slight overall decline. After 60 days, a moderate decrease in firmness was observed in most formulations compared to 30 days, with values ranging from 0.629 N (Cream-L@FN) to 0.729 N (Cream-L@ND), suggesting minor structural relaxation or reorganization within the emulsion network over time (Table 4). Despite this reduction, the creams maintained relatively consistent firmness, reflecting good physical stability throughout storage. Cohesiveness continued to decrease slightly, particularly in Cream-L@ND and Cream-L@FA, likely due to gradual water loss; however, Cream-L@FN maintained the highest cohesiveness (0.538), demonstrating superior internal structuring. Springiness remained relatively stable or even increased in certain formulations, particularly Cream-L@ND (1.012) and Cream-L@FN (1.042), indicating sustained elastic recovery and satisfactory spreadability after deformation.

Collectively, these findings confirm that all liposome-enriched creams retained acceptable textural characteristics after 60 days, with minimal deterioration in mechanical performance and good overall structural stability. These results suggest that while the creams retained their integrity, their internal matrix matured, becoming firmer but maintaining acceptable levels of elasticity and cohesiveness.

The spreadability test provides valuable insight into the ease of application of dermatocosmetic creams, a key factor in user comfort and product performance (Table 6).

Using the Ojeda–Arboussa method, the creams demonstrated a high degree of spreadability. These properties highlight the potential of these formulations for safe, effective, and consumer-acceptable cosmetic applications.

For all four liposome-based creams, the spreading surface area increases steadily as the amount applied increases (Table 6). This is typical behaviour for semi-solid products: the greater the amount applied, the thicker the film formed and the greater the surface area covered. The increases are approximately linear, but differ in amplitude between products. Cream-L@TR shows the highest spreading capacity. Over the entire range of measurements, Cream-L@TR consistently exceeds the other creams, with spreading surfaces of 2206 mm^2^ at 125 g and 5674 mm^2^ at 875 g. At the opposite pole, Cream-L@FN shows the lowest spreading capacity. Cream-L@FN has the smallest surface area of all the products when measured on almost all levels, with surface areas of 1963 mm^2^ at 125 g and 4071 mm^2^ at 875 g. Cream-base also demonstrated high spreadability, comparable to Cream-L@TR, indicating that the base formulation allows for efficient application and uniform film formation even in the absence of bioactive extracts. This behaviour can be attributed to the rheological properties of the excipients in the base, such as xanthan gum and Olliva emulsifier, which provide sufficient structural integrity while maintaining low resistance to flow under applied stress. The absence of densely loaded liposomes or particulate bioactives reduces internal resistance, facilitating deformation and spreading. Moreover, the semi-solid nature of the emulsion imparts shear-thinning behaviour, allowing the cream to spread easily during application. These observations are consistent with previous reports on nanomaterial- and extract-enriched cosmetic formulations, where excipient composition and matrix flexibility have a significant influence on spreadability [9,40,41].

The antioxidant activity of the samples was quantified using the DPPH radical scavenging assay and expressed as inhibition percentage and Trolox equivalents. All creams exhibit high inhibition percentage (>91%), indicating strong antioxidant activity. The values range narrowly from 1.725 to 1.777 mM/g, suggesting that all formulations have similar antioxidant capacities (Table 6). These results indicate the preservation of bioactive compounds post-formulation.

In terms of percentage inhibition, Cream-L@ND exhibited the highest antioxidant activity (93.79 ± 0.94%), with Cream-L@FN following closely behind (93.51 ± 0.94%). These values are almost identical, suggesting that the two formulas contain similar amounts of active compounds or ingredients with strong antioxidant properties. Cream-L@TR (91.72 ± 0.92%) and Cream-L@FA (91.08 ± 0.91%) recorded slightly lower values, but both still fall into the category of products with intense antioxidant activity (Table 7).

The results obtained are also supported by the determinations expressed in Trolox equivalents (TEAC), where the same order of antioxidant efficiency is observed. Cream-L@ND (1.777 ± 0.018 mM/g) and Cream-L@FN (1.772 ± 0.018 mM/g) have the highest values for equivalent antioxidant capacity, confirming the consistency of the data on radical inhibition. Cream-L@TR (1.737 ± 0.017 mM/g) and Cream-L@FA (1.725 ± 0.017 mM/g) have lower values that are still close, indicating comparable antioxidant activity that is slightly lower than that of the first two samples.

The Cream-base showed only modest activity (~11.36% inhibition), attributable to the minor antioxidant contribution from vitamin E and oils (Calendula officinalis oil, Argania spinosa oil, Helianthus annuus oil) in the excipient blend. Despite these baseline effects from the excipients, the 8- to 9-fold higher activity in the liposome-loaded creams clearly demonstrates that the predominant radical scavenging originates from the encapsulated grape pomace extracts (rich in phenolics like resveratrol, catechins, and proanthocyanidins) delivered via liposomes, rather than the other formulation components.

These findings align with the well-established radical-scavenging mechanisms of polyphenols and flavonoids, the major bioactive constituents of grape pomace extracts. These compounds exhibit antioxidant activity primarily through the donation of electrons or hydrogen atoms, which stabilizes reactive oxygen species (ROS) such as superoxide anions, hydroxyl radicals, and peroxyl radicals. By neutralizing these reactive species, polyphenols and flavonoids prevent oxidative damage to cellular lipids, proteins, and nucleic acids, thereby maintaining the structural and functional integrity of skin cells [42,43].

In addition to direct radical scavenging, polyphenols can chelate transition metal ions (e.g., Fe^2+^, Cu^2+^), which catalyze the formation of highly reactive radicals via Fenton-type reactions, reducing oxidative stress [44,45,46]. They may also modulate endogenous antioxidant defence systems by upregulating enzymes such as superoxide dismutase (SOD), catalase, and glutathione peroxidase, enhancing the skin’s intrinsic capacity to counteract oxidative damage [47,48].

The radical-scavenging effects of grape polyphenols can be conceptually compared to those of classical antioxidants such as vitamins C and E. Vitamin C primarily acts in the aqueous compartments of cells by donating electrons to neutralize free radicals, whereas vitamin E is lipid-soluble and protects cell membranes by intercepting lipid peroxyl radicals [49].

Polyphenols, depending on their structure, can act in both hydrophilic and lipophilic environments, allowing versatile protection across cellular compartments.

Encapsulation in liposomes enhances the antioxidant potential of grape pomace extracts by improving the stability and bioavailability of polyphenols and flavonoids. Liposomal encapsulation protects these compounds from degradation caused by environmental factors such as light, oxygen, and pH changes, while promoting deeper skin penetration. This targeted delivery ensures that bioactive molecules reach the dermal layers where oxidative stress is most pronounced, optimizing radical-scavenging and protective effects [50,51].

In general, the tested formulations demonstrate robust potential to counteract oxidative stress at the skin level. The higher concentrations of Trolox equivalents in Cream-L@ND and Cream-L@FN may indicate a greater amount of antioxidant ingredients or stronger synergy between the compounds in these formulations.

In conclusion, the analysis demonstrates that all evaluated dermatocosmetic creams exhibit strong antioxidant activity, with slight superiority observed in Cream-L@ND and Cream-L@FN. These results confirm the products’ potential to protect the skin against oxidative damage and support their use in formulations aimed at maintaining skin health and a youthful appearance.

### 3.3. Release Patterns of Polyphenols from Dermatocosmetic Creams

The four cream formulations (i.e., Cream-L@ND, Cream-L@FA, Cream-L@FN, and Cream-L@TR) exhibited a slow cumulative release of polyphenolic compounds. By 96 h, all formulations had approached their plateau values, with Cream-L@FA and Cream-L@TR achieving near-complete release (over 97%) and Cream-L@ND and Cream-L@FN reaching 91–92%. After 120 h, the cumulative release exceeded 95% for all samples. Overall, the data suggest that liposomal encapsulation within cream matrices can provide controlled, prolonged release of bioactive polyphenols.

**Figure 1:**
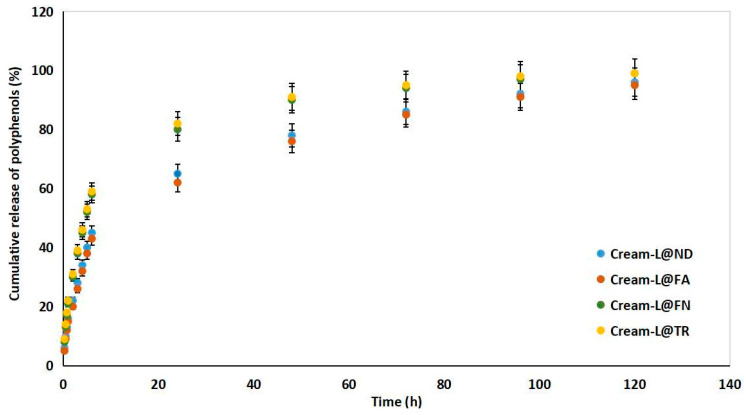
Controlled release of polyphenols from grape pomace extract-loaded liposomes enriched creams (i.e., Cream-L@ND, Cream-L@FA, Cream-L@FN, and Cream-L@TR).

It is evident that the Cream-L@TR and Cream-L@FN formulations have a faster onset and reach the plateau stage more quickly. In contrast, Cream-L@ND and Cream-L@FA have a slower release rate, although they also reach >95% at 120 h (Table 8 and Figure 1). The prolonged-release profiles of all formulas suggest the efficiency of liposomal encapsulation in controlling the release of polyphenols.

The release kinetics of polyphenols from proposed cream formulations were evaluated by fitting several mathematical models, including zero-order, first-order, Korsmeyer–Peppas, and Hixson–Crowell models, to identify the most accurate description of the release mechanism. Kinetic analyses were performed using KinetDS 3 software. The equations applied were as follows.

Zero-order kinetic

(3) $$M_{t}=K_{0}\cdot t$$

First-order kinetic

(4) $$M_{t}=100\cdot \left(1-e^{-kt}\right)$$

Korsmeyer–Peppas model:

(5) $$M_{t}=K_{p}\cdot t^{n}$$

Hixson–Crowell model:

(6) $$M_{t}^{\frac{1}{3}}=K_{HC}\cdot t+M_{0}^{\frac{1}{3}}$$

where $M_{t}$—amount of polyphenols released at time *t*; $K_{0}$—zero-order rate constant of the drug release rate; *k*—first-order rate constant; $K_{H}$—rate constant of the Higuchi model; $K_{p}$—Korsmeyer–Peppas release rate constant; *n*—exponential factor; *K_HC_*—Hixson–Crowell release rate constant.

The most appropriate kinetic model was determined based on the correlation coefficient (R^2^), root mean square error (RMSE), and Akaike information criterion (AIC). A suitable model was considered one that demonstrated an R^2^ value close to 1.0, along with low RMSE and AIC values, indicating accurate and reliable prediction of polyphenol release behaviour.

Table 9 shows R^2^, RMSE, and AIC values for the fitting models. The polyphenol release profiles from dermatocosmetic products were best described by the Korsmeyer–Peppas model for all formulations. This is evidenced by the highest correlation coefficients (R^2^) and the lowest RMSE and AIC values compared to the other models. Specifically, creams containing liposomes loaded with red and white grape pomace extract showed R^2^ values of 0.953, 0.921, 0.955, and 0.926, respectively, indicating a strong fit to the Korsmeyer–Peppas equation. In contrast, the zero-order and Hixson–Crowell models showed moderate correlation (R^2^ ranging from 0.575 to 0.831) with higher RMSE and AIC values, and the first-order model exhibited the poorest fit (R^2^ between 0.490 and 0.562).

Table 10 presents the Korsmeyer–Peppas release mechanism parameters and highlights that all formulations had the exponent values (n) below 0.5, suggesting Fickian diffusion as the primary release mechanism.

**Figure 2:**
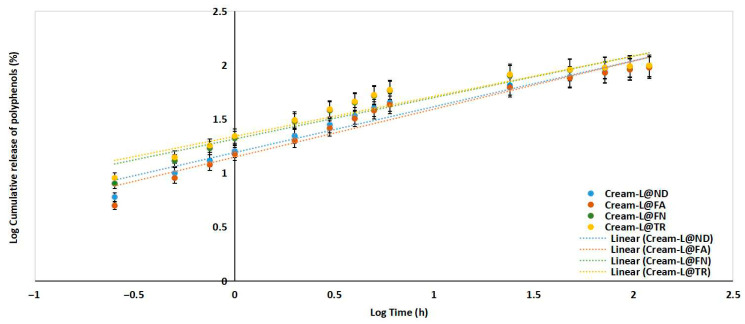
Cumulative release of polyphenols from grape pomace extract-loaded liposomes enriched creams (i.e., Cream-L@ND, Cream-L@FA, Cream-L@FN, and Cream-L@TR) according to Korsmeyer–Peppas kinetic model.

Based on the K_p_ values (Table 10 and Figure 2), the Cream-L@TR and Cream-L@FN formulations release the active ingredient faster than the Cream-L@ND and Cream-L@FA formulations. All creams demonstrate a Fickian diffusive release mechanism (n < 0.5). The differences in K_p_ values demonstrate the effect of composition on release rate, despite the mechanism remaining the same.

## 4. Conclusions

The red and white grape pomace extracts from the local Fetească Neagră and Negru de Drăgășani varieties, and Fetească Albă and Tămâioasă Românească varieties, respectively, are embedded in liposomes and harmonized with a series of natural essential oils, such as *Calendula officinalis*, *Argania spinosa*, and *Helianthus annuus*, together with xanthan gum, Olliva emulsifier, and vitamin E. The cosmetic formulations moisturize the skin, reduce wrinkles, and have an anti-ageing effect, mainly due to the active principles of the natural ingredients used. Extracts obtained using eco-friendly methods exhibited high phenolic compound content and strong antioxidant activity. Liposomal encapsulation protected sensitive compounds, enhanced stability, and allowed controlled release. The liposomes had a suitable nanometric size (166–184 nm), a uniform distribution (PDI < 0.35), and a good encapsulation efficiency (60–66%). They were also compatible with topical application. When incorporated into O/W creams, they produced stable formulations for 60 days without any organoleptic changes or phase separation, and the pH values stayed within the physiological range. Texture analysis and spreading testing showed that all new formulations had physical properties suitable for dermatocosmetic use, demonstrating good homogeneity, firmness, and elasticity. The high antioxidant activity (>90%) confirms that the bioactive compounds are preserved after formulation. Release studies indicated sustained, near-complete polyphenol release (≥95% at 120 h), highlighting the role of liposomal encapsulation in controlling diffusion.

Overall, the results demonstrate the feasibility, sustainability, and efficiency of valorising pomace in dermatocosmetic products, providing a concrete example of transforming agro-industrial waste into a high-value ingredient. These formulations have real potential for application in the cosmetics industry, as they help reduce environmental impact while enabling the development of natural products with proven biological efficacy.

## 5. Patents

Radulescu C., Olteanu R.L., Pavaloiu R.D., Sha’at F., Nechifor (Tudorache) Mihaela, Cremă Dermatocosmetică pe Bază de Lipozomi cu Extract de Tescovină din Struguri Roșii (Dermatocosmetic Cream Based on Liposomes with Red Grape Seed Extract) patent application A/00030/2026 (national patent OSIM).

## Figures and Tables

**Table 1:** Composition of dermatocosmetic formulations enriched with liposomes with red/white grape pomace extracts and natural components.

Component	Cream Enriched with Liposomes			
	Cream-L@ND	Cream-L@FA	Cream-L@FN	Cream-L@TR
Xantan Gum	0.5%	0.5%	0.5%	0.5%
Prolpylenglycol	8%	8%	8%	8%
*Calendula officinalis* oil	5%	5%	5%	5%
*Argania spinosa* oil	5%	5%	5%	5%
*Helianthus annuus* oil	5%	5%	5%	5%
Vitamin E	0.5%	0.5%	0.5%	0.5%
Cetearylic Alcohol	3%	3%	3%	3%
Olliva Emulsifier	6%	6%	6%	6%
L@ND	2%	-	-	-
L@FA	-	2%	-	-
L@FN	-	-	2%	-
L@TR	-	-	-	2%
Purified water	Up to 100%	Up to 100%	Up to 100%	Up to 100%

**Table 2:** Characteristics of liposomes loaded with red and white pomace extracts.

Characteristics	Liposomes Loaded with Red and White Pomace Extracts			
	L@ND	L@FN	L@FA	L@TR
EE [%]	65.62 ± 0.06	65.12 ± 0.06	60.20 ± 0.05	60.12 ± 0.05
Size [nm]	184.2 ± 2.40	177.8 ± 1.30	167.3 ± 0.90	166.3 ± 2.50
PDI	0.301 ± 0.02	0.331 ± 0.04	0.322 ± 0.06	0.350 ± 0.06

**Table 3:** Characteristics of dermatocosmetic formulations based on liposomes loaded with red and white pomace extracts.

Characteristics	Cream-L@ND	Cream-L@FA	Cream-L@FN	Cream-L@TR
Organoleptic evaluation-after 24 h	Appearance: Homogeneous	Appearance: Homogeneous	Appearance: Homogeneous	Appearance: Homogeneous
	Colour: pearl yellowish-white	Colour: pearl yellowish-white	Colour: pearl yellowish-white	Colour: pearl yellowish-white
	Smell: No odour	Smell: No odour	Smell: No odour	Smell: No odour
pH-after 24 h	5.01 ± 0.01	5.08 ± 0.01	5.09 ± 0.01	5.07 ± 0.006
	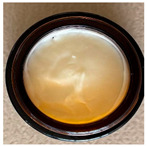	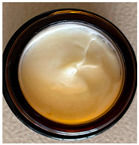	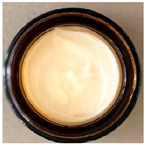	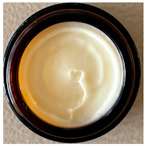
Organoleptic evaluation-after 30 days (storage at 4–8 °C)	Appearance: Homogeneous	Appearance: Homogeneous	Appearance: Homogeneous	Appearance: Homogeneous
	Colour: pearl yellowish-white	Colour: pearl yellowish-white	Colour: pearl yellowish-white	Colour: pearl yellowish-white
	Smell: No odour, No signs of phase separation, sedimentation, or texture alteration	Smell: No odour, No signs of phase separation, sedimentation, or texture alteration	Smell: No odour, No signs of phase separation, sedimentation, or texture alteration	Smell: No odour, No signs of phase separation, sedimentation, or texture alteration
pH-after 30 days (storage at 4–8 °C)	5.12 ± 0.04	5.15 ± 0.03	5.24 ± 0.04	5.22 ± 0.04
	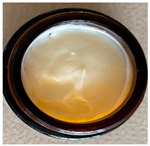	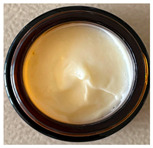	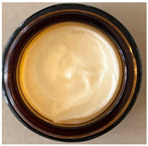	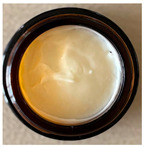
Organoleptic evaluation-after 60 days (storage at 4–8 °C)	Appearance: Homogeneous	Appearance: Homogeneous	Appearance: Homogeneous	Appearance: Homogeneous
	Colour: pearl yellowish-white	Colour: pearl yellowish-white	Colour: pearl yellowish-white	Colour: pearl yellowish-white
	Smell: No odour, No signs of phase separation, sedimentation, or texture alteration	Smell: No odour, No signs of phase separation, sedimentation, or texture alteration	Smell: No odour, No signs of phase separation, sedimentation, or texture alteration	Smell: No odour, No signs of phase separation, sedimentation, or texture alteration
pH-after 60 days (storage at 4–8 °C)	5.35 ± 0.03	5.36 ± 0.02	5.35 ± 0.03	5.37 ± 0.02

**Table 4:** Composition and characteristics of cream-base formulation (after 24 h).

Component	Cream-Base	Organoleptic Evaluation	pH	
Xantan Gum	0.5%	Appearance: Homogeneous Colour: pearl yellowish-white Smell: No odour	5.25 ± 0.01	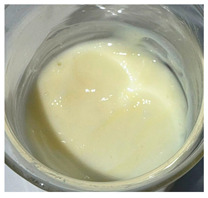
Prolpylenglycol	8%			
*Calendula officinalis* oil	5%			
*Argania spinosa* oil	5%			
*Helianthus annuus* oil	5%			
Vitamin E	0.5%			
Cetearylic Alcohol	3%			
Olliva Emulsifier	6%			
L@ND	-			
L@FA	-			
L@FN	-			
L@TR	-			
Purified water	Up to 100%			

**Table 5:** TPA profile of dermatocosmetic creams on 24 h, 30 days, and 60 days.

Characteristics	Cream-L@ND	Cream-L@FA	Cream-L@FN	Cream-L@TR
**24 h**				
Firmness (hardness)	0.453 ± 0.012 N	0.500 ± 0.011 N	0.394 ± 0.013 N	0.457 ± 0.011 N
Cohesiveness	0.541 ± 0.019	0.564 ± 0.018	0.598 ± 0.019	0.577 ± 0.059
Springiness	0.975 ± 0.025	0.997 ± 0.026	0.922 ± 0.026	0.790 ± 0.045
**30 days**				
Firmness (hardness)	0.753 ± 0.052 N	0.801 ± 0.012 N	0.602 ± 0.014 N	0.584 ± 0.012 N
Cohesiveness	0.461 ± 0.079	0.476 ± 0.076	0.498 ± 0.019	0.474 ± 0.039
Springiness	0.895 ± 0.095	0.895 ± 0.045	0.872 ± 0.026	0.838 ± 0.085
**60 days**				
Firmness (hardness)	0.729 ± 0.013 N	0.712 ± 0.010 N	0.629 ± 0.013 N	0.712 ± 0.011 N
Cohesiveness	0.438 ± 0.018	0.454 ± 0.017	0.538 ± 0.010	0.450 ± 0.039
Springiness	1.012 ± 0.026	0.854 ± 0.026	1.042 ± 0.016	0.846 ± 0.025

**Table 6:** Spreadability values (24 h).

Spreading Surface Area [mm^2^]	Cream-L@ND	Cream-L@FA	Cream-L@FN	Cream-L@TR
initial 125 g	2123.7148 ± 63.71	2042.8189 ± 61.28	1963.4938 ± 58.90	2206.1816 ± 66.19
175	2463.0066 ± 73.89	2290.2191 ± 68.71	2206.1816 ± 66.19	2642.0772 ± 79.26
225	2733.9687 ± 82.02	2551.7565 ± 76.55	2463.0066 ± 73.89	3318.3044 ± 99.55
275	3318.3044 ± 99.55	2922.4641 ± 87.67	2827.4310 ± 84.82	3525.6494 ± 105.77
375	3848.4478 ± 115.45	3019.0680 ± 90.57	2922.4641 ± 87.67	3959.1888 ± 118.78
625	4071.5006 ± 122.15	3739.2775 ± 112.18	3631.6780 ± 108.95	4417.8609 ± 132.54
875	5152.9930 ± 154.59	4185.3833 ± 125.56	4071.5006 ± 122.15	5674.4969 ± 170.23

**Table 7:** Antioxidant activity of dermatocosmetic formulations based on liposomes loaded with red and white pomace extracts.

Sample Code	% Inhibition (Mean ± SD)	Trolox Eq. [mM/g] ± SD
**Cream-** **L@ND**	93.79 ± 0.94	1.777 ± 0.018
**Cream- L@FA**	91.08 ± 0.91	1.725 ± 0.017
**Cream- L@FN**	93.51 ± 0.94	1.772 ± 0.018
**Cream- L@TR**	91.72 ± 0.92	1.737 ± 0.017
**Cream-base**	11.36 ± 0.77	0.183 ± 0.015

**Table 8:** Cumulative release of polyphenols from prepared cosmetic formulations.

Time [h]	Cream-L@ND [%]	Cream-L@FA [%]	Cream-L@FN [%]	Cream-L@TR [%]
0.25	6	5	8	9
0.5	10	9	13	14
0.75	13	12	17	18
1	16	15	21	22
2	22	20	30	31
3	28	26	38	39
4	34	32	45	46
5	40	38	52	53
6	45	43	58	59
24	65	62	80	82
48	78	76	90	91
72	86	85	94	95
96	92	91	97	98
120	96	95	99	99

**Table 9:** Correlation coefficient (R^2^), root mean square error (RMSE), and Akaike information criterion (AIC) of the fitted experimental data.

Kinetic Model	Model Coefficients	Dermatocosmetic Formulation			
		Cream-L@ND	Cream-L@FA	Cream-L@FN	Cream-L@TR
*Zero-order*	R^2^	0.818	0.831	0.715	0.708
	RMSE	13.28	12.80	17.24	17.45
	AIC	113.36	112.33	120.68	121.00
*First-order*	R^2^	0.562	0.562	0.490	0.495
	RMSE	22.57	22.87	24.78	24.60
	AIC	128.21	128.59	130.83	130.62
*Korsmeyer–Peppas*	R^2^	0.955	0.953	0.921	0.926
	RMSE	9.13	9.25	13.05	12.66
	AIC	102.86	103.23	112.88	112.02
*Hixson–Cromwell*	R^2^	0.663	0.670	0.575	0.575
	RMSE	17.51	17.31	20.77	20.84
	AIC	121.11	120.78	125.89	125.99

**Table 10:** Parameters of the Korsmeyer–Peppas model.

Formulation	K_P_ (Release Constant)	n (Exponent)
**Cream-L@ND**	15.4974	0.4250
**Cream-L@FA**	14.0616	0.4496
**Cream-L@FN**	20.6852	0.3842
Cream-L@TR	21.8876	0.3712

## Data Availability

The original contributions presented in this study are included in the article; further inquiries can be directed to the corresponding author.
